# Recent Progress and Challenges in Hollow Fiber Membranes for Wastewater Treatment and Resource Recovery

**DOI:** 10.3390/membranes11110839

**Published:** 2021-10-29

**Authors:** Rosmawati Naim, Goh Pei Sean, Zinnirah Nasir, Nadzirah Mohd Mokhtar, Nor Amirah Safiah Muhammad

**Affiliations:** 1 Faculty of Chemical and Process Engineering Technology, College of Engineering Technology, Universiti Malaysia Pahang, Lebuhraya Tun Razak, Kuantan 26300, Pahang, Malaysia; zinnirah68@gmail.com; 2 Advanced Membrane Technology Research Centre (AMTEC), School of Chemical and Energy Engineering, Faculty of Engineering, Universiti Teknologi Malysia, Johor Bahru 81310, Johor, Malaysia; peisean@petroleum.utm.my; 3 Faculty of Civil Engineering Technology, College of Engineering Technology, Universiti Malaysia Pahang, Lebuhraya Tun Razak, Kuantan 26300, Pahang, Malaysia; nadzirah@ump.edu.my (N.M.M.); noramirahsafiah@yahoo.com (N.A.S.M.)

**Keywords:** hollow fiber membrane, wastewater treatment, resource recovery

## Abstract

Membrane processes have been extensively employed in diverse applications, specifically in industrial wastewater treatment. The technological development in membrane processes has rapidly advanced and accelerated beyond its common principle and operation. Tremendous efforts have been made in the advancement of membrane materials, fabrication method, membrane modification and integration with other technologies that can augment the existing membrane processes to another level. This review presents the recent development of hollow fiber membranes applied in wastewater treatment and resource recovery. The membrane working principles and treatment mechanism were discussed thoroughly, with the recent development of these hollow fiber membranes highlighted based on several types of membrane application. The current challenges and limitations which may hinder this technology from expanding were critically described to offer a better perspective for this technology to be adopted in various potential applications.

## 1. Introduction

Global water scarcity and resource shortage resulting from the exponential population growth, industrialization and urbanization represent great challenges for the humans of the 21st century [1]. The global increase in the human population has led to the growth of various industries. The requirement for an excess supply of water and the generation of high effluent quality with proper treatment technologies has become a necessary aspect. Despite the harmful effect on the ecological environment and human health, the valuable and rare resource elements in wastewater have considerable market value. According to Barros et al. [2], the rare earth resource elements lanthanum (La), cerium (Ce), yttrium (Y), terbium (Tb), praseodymium (Pr) and europium (Eu) can be recycled from the wastewater and reused in production cycle. These recycled rare earths have attractive value in the market, ranging from USD 4.50/kg up to USD 95/kg [3]. Although it is still a new field, resource recovery from wastewater will draw more attention with the increase in the worldwide population and resource shortages.

Various strategies have been implemented to address the increasing demands on clean water and resources. The recovery of valuable materials from wastewater fits the principle of circular economy and sustainable use of resources, but contaminants in the wastewater are still a major obstacle that needs to be treated. Membrane separation processes serve as an important innovation in treating wastewater and sustaining resource recovery in many industries. Principally, the membrane separation process involves the separation of mixture in the feed stream when passing through a semi-permeable barrier. The membrane, which is known as the heart of the entire membrane process, controls the permeation of specific type of chemical species through its cross-sectional structure to allow one component of a mixture to permeate the membrane freely while hindering the permeation of other components in various applications, such as wastewater treatment [4].

Conventionally, wastewater treatment and resource recovery have been accomplished through approaches such as electrochemical treatment, precipitation [5], ion exchange [6], adsorption [7], flotation [8], and coagulation/flocculation [9]. Membrane separation has gained increasing attention as an alternative to these conventional approaches due to its reliability and efficiency in treating wastewater and the simultaneous nutrient recovery during the wastewater treatment process. Membrane technology demonstrates various advantages, such as stable effluent quality, small footprint, simple system design and relatively low energy consumption compared to other conventional counterparts [4,10]. For example, compared to conventional distillation, which separates components or substances from liquid mixture by using selective boiling and condensation point, the membrane separation process allows the separation of a mixture of different molecular sizes with much less heat and energy. The modularity of the membrane system is another advantage that can be utilized for small operation areas, such as oil and gas platforms and remote areas. With these benefits, the membrane separation process offers huge potential for upscaling and high-performance operation.

Direct membrane filtration has shown great potential in wastewater treatment and resource recovery in terms of its excellent treated water quality, efficient nutrient recovery, and sustainable operation, especially in some situations in which biological treatment is not feasible [11]. The biological-based wastewater treatment process may not be feasible for all types of wastewater compositions (e.g., low-strength wastewater) due to its relatively low treatment efficiency and high dependence on environmental factors, such as temperature, variation of feed composition and oxygen level [12,13]. It is also noted that most biological treatment processes require intensive energy consumption, especially for aeration. Additionally, the organics/nutrients in wastewater are converted into CO_2_, producing a large amount of greenhouse gas emission [14,15].

Membranes used for wastewater treatment are typically fabricated into 2 configurations, i.e., flat sheet and hollow fiber in which the hollow fiber provides more effective surface area than the flat sheet type. Hollow fiber membrane can be used in all types of filtrations, ranging from microfiltration (MF) to reverse osmosis (RO). Hollow fiber filtration works on the same principle as tubular and capillary configurations, but the flexibility of the membrane can be enhanced with smaller tube diameter. Common applications of hollow fiber membranes in wastewater treatment and resource recovery includes ultrafiltration (UF), forward osmosis (FO), pressure retarded osmosis (PRO), membrane distillation (MD) and membrane contactor (MC). 

Several reviews have been reported on the treatment for wastewater and resource recovery via membrane technology. Goh et al. [16] elaborated on the potential of various membrane-based separation processes for saline wastewater treatment and resource recovery. Meanwhile, Rongwong and Goh [17] discussed the recovery of industrial wastewater by hydrophobic membrane contactor. In this paper, the primary focus is to discuss and gather data on the potential and effectiveness of hollow fiber membranes in treating the wastewater and resource recovery via various membrane applications. Finally, the challenges and future direction of hollow fiber in wastewater and resource recovery will be discussed.

## 2. Common Types of Membrane Processes for Wastewater Treatment

Hollow fiber membrane processes are viable for a wide range of wastewater treatment processes, including pressure-driven processes, such as UF and RO, and osmotically driven processes, such as FO and PRO, as well as MD and HFMC. Table 1 describes the applications of hollow fiber membrane in various wastewater treatments.

### 2.1. Ultrafiltration

**Figure 1:**
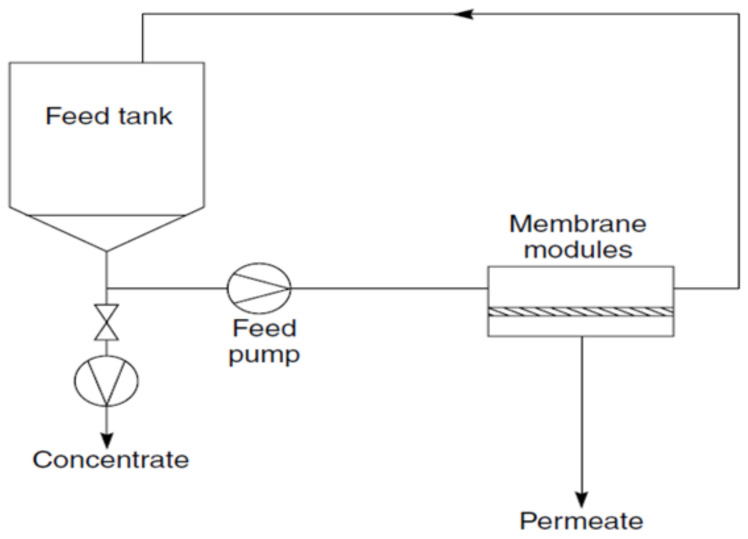
Schematic flow of a batch ultrafiltration process.

**Figure 2:**
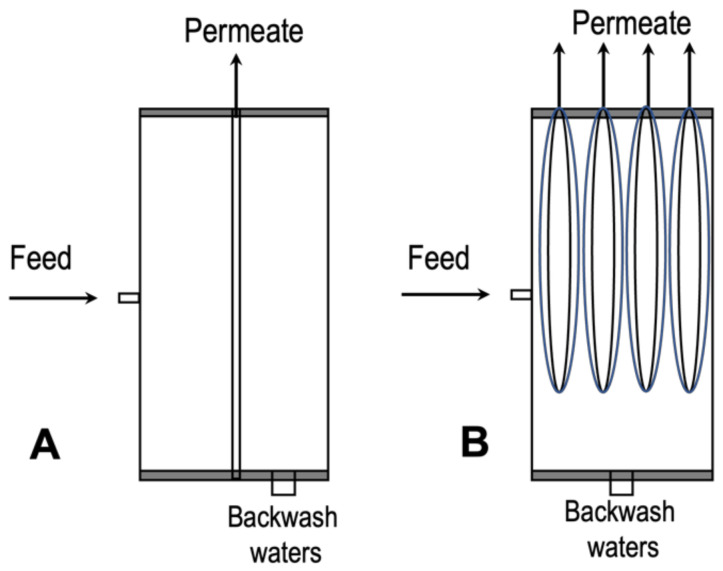
Sectional drawings and pictures of the filtration modules unit (**A**) and (**B**) [18].

UF with hollow fiber membrane configuration has been widely used for wastewater treatment to remove suspended matter, colloidal particles, bacteria or viruses from a wide range of waste streams, including domestic and industrial effluents [18]. The simplest type of UF system is a batch unit, as shown in Figure 1. In such unit, a limited volume of the feed solution is circulated through a module at a high flow rate. The process continues until the required separation is achieved, after which the concentrate solution is drained from the feed tank, and the unit is ready to treat the second batch of solution [29]. Vroman et al. [18] utilized PVDF hollow-fiber membranes for fouling removal via an ultrafiltration process of domestic wastewater treatment collected from Veolia Brax, France. Two filtration units were designed in the lab, which are filtration unit A and filtration unit B as shown in Figure 2. Both were working at constant pressure in dead-end and outside-in mode. The flow was reversed to perform a backwash at constant pressure where the removal of cake and membrane permeability was measured. They reported that more than 60% of the humic cake was removed in specific conditions where the membrane experienced a deformation exceeding 15%.

Alresheedi et al. investigated the influence of support media on fouling in a bench-scale submerged hollow fiber membrane UF system made of high-density polyethylene (HDPE) for the separation of synthetic wastewater comprised of different organic and inorganic matter content. The study revealed that membrane fouling in the system with support media exhibited lower fouling propensity compared to the membrane system without support media [19]. Ong et al. [20] prepared polyvinylidene fluoride (PVDF) hollow fiber UF membranes incorporated with different titanium dioxide (TiO_2_) nanomaterials with the presence of polyvinylpyrrolidone as an additive. The membrane performances were characterized in terms of pure water flux, permeate flux and oil rejection, while the membrane morphologies were analyzed using a scanning electron microscope and atomic force microscope. The results showed that when 2 wt.% TiO_2_ was incorporated into PVDF membranes, the optimized permeate flux and oil rejection have a value of 70.48 L/m^2^ h (±1.41) and 99.7% (±0.3), respectively. The results concluded that the composite PVDF membrane showed better performance in treating oily solution compared to that without TiO_2_.

### 2.2. Forward Osmosis and Pressure Retarded Osmosis 

**Figure 3:**
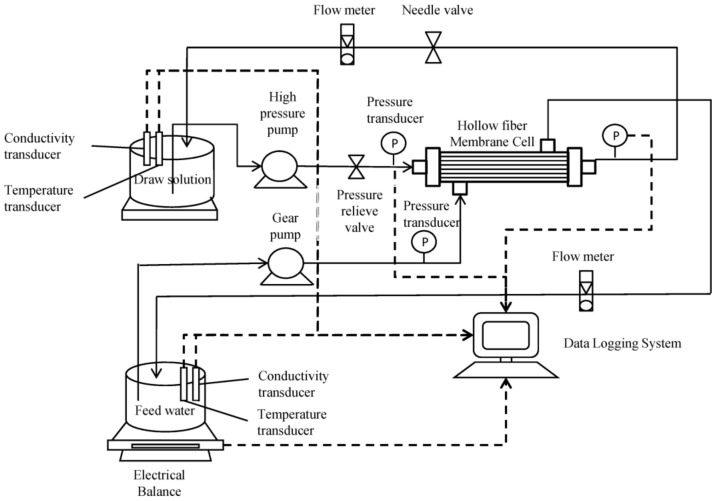
Schematic diagram of bench-scale PRO system [27].

The application of osmotically driven membrane processes (ODMPs), including FO and PRO, for wastewater treatment and nutrient recovery have received increasing attention. These processes have been known as another promising solution in providing a sustainable answer against water and energy shortage issues. By utilizing the osmotic pressure difference between two water bodies, i.e., feed (low salinity) and draw solution (high salinity), across a semipermeable membrane, such as a hollow fiber membrane, osmotically driven processes do not require external hydraulic pressure for their operations. FO and PRO are known as highly suitable technologies to facilitate the recovery of clean water, nutrients and energy from wastewater [30]. There has been a significant increase in the interest of FO and PRO recently because of the potential benefits, including low-energy stabilization of wastewater and resource recovery. A high level of dissolved contaminants could be rejected to generate clean water from the draw agent when a draw solute recovery process is included, leading to further opportunities for water reuse. Zhao et al. investigated the fouling behavior and chemical cleaning of FO membranes for treating produced/process water (PPW) from a real gas field using thin-film composite polyethersulfone (PES) hollow fiber membranes. The results showed that the flux profile of the FO hollow fiber membrane during the treatment achieved a 50% volume reduction with an average water flux of 15.6 L/m^2^ h [22]. PRO based on hollow fiber membranes is also widely used in wastewater treatment. PRO is an intermediate osmosis process between the FO and RO; the hydrostatic pressure of the draw solution is lower than the osmotic pressure difference across the membrane. This can cause the water to permeate from the freshwater side to the saltwater side [27]. A schematic diagram of the bench-scale PRO setup is shown in Figure 3. 

### 2.3. Membrane Distillation 

**Figure 4:**
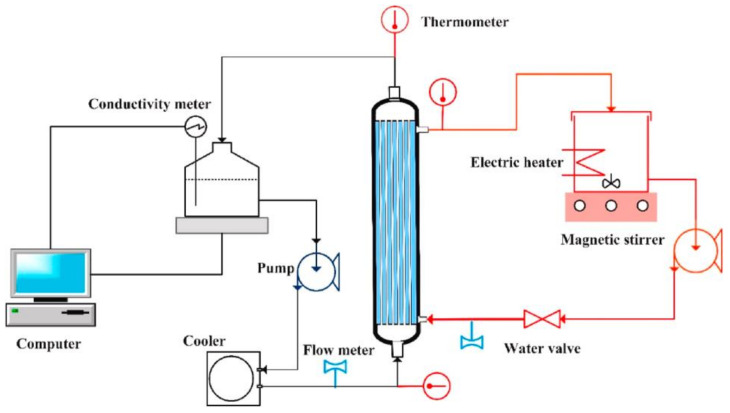
Schematic drawing of the apparatus for direct contact membrane distillation (DCMD) [25].

MD is another wastewater treatment option. The process relies on a micro-porous hydrophobic membrane to create a gas-liquid interface for the separation of two solutions at different temperatures. As the separation efficiency strongly depends on the membrane structure and volatility of the components to be separated, MD can achieve high rejection of non-volatile organic substances at operating pressure lower than conventional RO processes. The hydrophobic membrane characteristics play an important role in the permeate flux enhancement, which involve coupled vapor and heat transportation where high vapor transfer rate and low conductive heat transfer rate are preferred as a property for the membranes to be employed in DCMD. The schematic diagram of the apparatus for direct contact membrane distillation (DCMD) is shown in Figure 4. DCMD is an attention grabber due to its capability to utilize low-grade heat, such as geothermal resources, solar energy, waste heat streams and subterranean heat. DCMD has been extensively used in desalination for high saline feed water, reclamation of industrial process water, juice concentration, purification of groundwater and removal of micropollutants from drinking water. It operates based on the principle of vapor/liquid equilibrium. Theoretically, the salt rejection is 100%, and it has been extensively reported for the desalination of hypersaline wastewater [31,32]. However, DCMD is still challenged by factors including lower water flux than the conventional reverse osmosis and both membrane fouling and wetting phenomenon in long-term DCMD operation, which impact large-scale industrial application [33]. Cho et al. [23] studied the effect of pretreatment and operating conditions on the fouling behaviors of membranes using hollow fiber membranes made of PVDF, polyethylene (PE), and polypropylene (PP) and compared them in a laboratory-scale direct contact membrane distillation (DCMD) system on the feedstock obtained from real shale gas wastewater from oil and gas field in Texas, United States. The results showed that the flux reduction ratio ranges from 13.6% to 27.7% to achieve a 50% recovery ratio without pretreatment.

**Figure 5:**
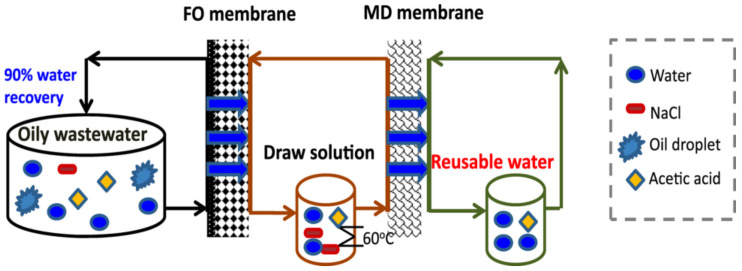
Schematic diagram of combination of forward osmosis (FO) and membrane distillation (MD) for wastewater treatment [21].

Jia et al. [24] examined the separation of cobalt ions (Co^2+^) by vacuum membrane distillation (VMD) process with commercial PP hollow fiber membranes using simulated radioactive wastewater. The Pearson correlation analysis was done to evaluate the significance of different operating parameters to the permeate flux with the results of the removal efficiency over 99.67% when influent Co(II) concentration was about 10 mg/L. Another crosslinked PVDF-based hydrophilic–hydrophobic dual-layer hollow fiber membrane was fabricated by Zou et al. [25] for the DCMD process using simulated seawater and actual oilfield produced water as the feed solutions. The results showed that almost 100% of permeate water recovery and 99.9% of salt rejection came out of the process. The schematic diagram of the combination of FO and MD setup for wastewater treatment are shown in Figure 5. 

### 2.4. Hollow Fiber Membrane Contactor

**Figure 6:**
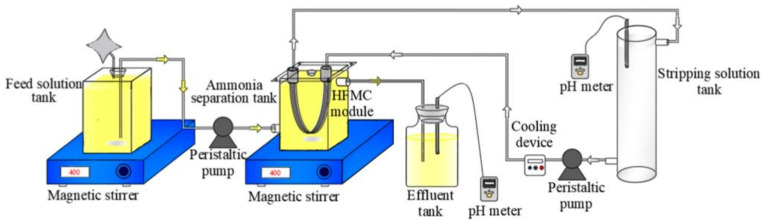
Schematic diagram of the hollow fiber membrane contactor set-up [1].

Nowadays, renewable and environmentally friendly resource recovery is considered an alternative to the shortage of resources. This has received much attention lately. Resource recovery (e.g., ammonia, nitrogen gas and carbon dioxide) can be generated through wastewater treatment using various technologies. To recover the resources from wastewater, a hollow fiber membrane contactor (HFMC) has been considered as one of the most efficient technologies to recover resources from various wastewater sources. The schematic diagram of an HFMC process set-up is shown in Figure 6. HFMC has many advantages, including high efficiency, easy operation under regular pressures and lower cost of operations as compared to air stripping [34,35]. In addition, the feed and stripping solution are separated by the membrane in HFMC systems; hence, the problems of flooding, diversion and foaming in the conventional air stripping process can be prevented effectively [36]. The performance of the membrane contactor solely depends on the membrane hydrophobicity properties, which, in the long run, can deteriorate due to a wetting problem. Therefore, many efforts are focused on improving the membrane properties and maintaining the robustness of the membrane under severe conditions in wastewater treatment. 

## 3. Recent Development in Hollow Fiber Membrane in Wastewater Treatment

Attempts have been made to increase the removal performance via selection of new membrane materials, modification of membrane for the improvement of membrane properties and integration of membrane processes with other conventional or advanced processes. With the constant efforts, membrane technology has demonstrated its capability to solve numerous process uncertainties and constraints in diverse industry applications.

In the case of ammonia-containing wastewater, chemical industries, such as fertilizer plants and plastics producers, utilize this compound as a cleaning and bleaching agent. The discharge of ammonia from the wastewater treatment plant and industrial processes has become a threat to the environment and living things as it can cause eutrophication, offensive odor contamination and hinder the disinfection of the water supply. The recovery of ammonia has the potential to be applied in electrical power generation and the field of carbon-free energy storage. For example, Sancho et al. [26] attempted to recover ammonia from domestic wastewater through the integration of the extraction process via modification of natural zeolite as a pretreatment process followed by liquid–liquid hollow fiber membrane contactor based on propylene membrane. They reported promising results in which nitric and phosphoric acids in HFMC contributed to more than 95% of total nitrogen recovery capacity when excess free acid was present in the stripping stream. Hence, diminishing the usage of low-cost acids, such as H_2_SO_4_ and HCl, as ammonia collectors produces value-added waste as fertilizers.

Zhang et al. [1] developed a submerged PP HFMC to recover ammonia from human urine to obtain compound nitrogen and phosphorous (NP) fertilizers. The study investigated the ammonia capture performance, water vapor transmembrane performance, ion rejection performance and the liquid fertilizer components. They used synthetic hydrolyzed human urine with pH of 9.10 ± 0.3 as the feed waste in this work. The ammonia recovery efficiency was varied from 88.47% to 90.90% using 1–4 v mol/L as H_3_PO_4_, the stripping solution. They also forecasted an economic profit of USD 7.089/L from harvesting liquid N-P fertilizer from human urine using this HFM system. 

Damtie et al. [28] employed a liquid/liquid hydrophobic PVDF HFMC at ambient temperature and human urine pH ~ 9.7 to recover ammonium fertilizers from domestic wastewater. As a result, high-quality liquid ammonium sulfate, ammonium monophosphate/diphosphate and ammonium nitrate fertilizer were produced. Another effort by Ma et al. [37] used electrospun hollow fiber membranes for removal of ammonia nitrogen wastewater to demonstrate that home-fabricated PVDF-HFP hollow fiber membrane outperformed the commercial PP membrane in terms of contact angle and membrane mass transfer coefficient due to the higher porosity and stronger hydrophobicity from the water of PVDF-HFP over PP materials. In terms of selectivity performance, the PVDF-HFP membrane produced an overall mass transfer coefficient (KOE) of 1.35 × 10^−5^ m/s, and SNH_3_/H_2_O is 7.58 when the pH is 11, which is much higher than that of the commercial membrane. 

As for membrane modification effort, Lim et al. [38] fabricated a defect-free outer-selective hollow fiber (OSHF) thin-film composite (TFC) membrane for FO application. This selective PES HFM was coated with a polyamide (PA) layer via modified vacuum-assisted interfacial polymerization (VAIP) in which the excess substrate layer on the outer HF membrane was effectively removed by vacuum suction to the bore side of the membrane. They reported the membrane performance in terms of water flux of 30.2 L/m^2^ h and a specific reverse solute flux of 0.13 g/L using 1 M of NaCl and DI water as draw and feed solution. When tested using silica-alginate solution, the membrane demonstrated high fouling resistance with higher cleaning efficiency. It is believed that this method of membrane modification is potentially suitable for emerging FO applications, specifically in the area of submerged aerobic or anaerobic osmotic membrane bioreactors (OMBRs) and fertilizer drawn OMBR hybrid systems, in which the concern of membrane fouling, low water flux and membrane modulation can be minimized.

In the development of an osmotically driven process, Chou et al. [27] fabricated TFC FO PES hollow fiber membranes followed by PRO using polyamide hollow fiber membranes for seawater and wastewater brine to harvest excellent separation properties and high-water flux. The TFC PRO hollow fiber membranes have a water permeability (A) of 9.22 × 10^−12^ m/(s Pa), salt permeability (B) of 3.86 × 10^−8^ m/s and structural parameter (S) of 4.6 × 10^−4^ m. NaCl was used as the solute to simulate the osmotic potential of both the draw solution (0.5 M, 0.75 M and 1.0 M) and the feed water (10 mM, 40 mM and 80 mM). Their findings illustrated the increase in water flux by 23% (40.3 L/m^2^ h), while PRO managed to withstand hydrostatic pressure as high as 9 bar with a power density of 10.6 W/m^2^ with simulated seawater brine (1.0 M NaCl) and wastewater brine (40 mM NaCl) [24]. Table 2 tabulates the recent development of hollow fiber membrane used for wastewater treatment and resource recovery. 

In food industries, attempts have been initiated to utilize its waste and turn it into a more useful product. For example, Li et al. [44] attempted to recover potato protein from potato starch wastewater. Most potato protein recovery is concentrated on the flat sheet or tubular UF and RO membranes. It does not indicate in-depth discussion on the fouling behavior in such a system. Their work focused on the preparation of composite polysulfone membrane via interfacial polymerization of piperazine (PIP) and trimesoyl chloride (TMC) on the lumen side membranes. The in-house membrane showed protein rejection of 85.6 and 92.1% for UF and NF membrane, while COD rejections reached up to 57.3% and 86.8% for UF and NF. However, the prominent bulges were observed on the membrane surface after the separation process, confirming that the membrane suffers predictable fouling. Recovery of membrane water flux can be obtained by cleaning and pure water washing with alkaline solution. The UF and NF flux recovery reached up to 93.5 and 84.5%, respectively.

**Figure 7:**
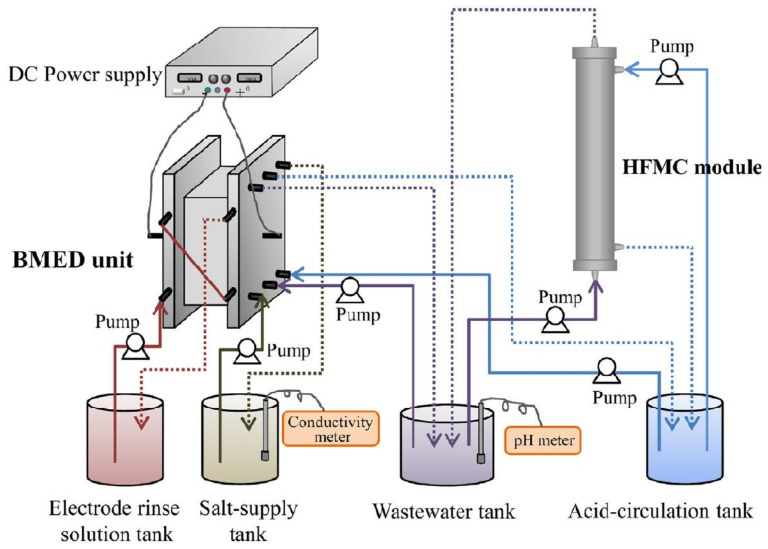
Schematic experimental setup of bipolar membrane electrodialysis and hollow fiber membrane contactor for ammonia capture from wastewater [39].

Yan et al. [39] have reported a hybrid system consisting of bipolar membrane electrodialysis (BMED) and HFMC for ammonia capture from wastewater (Figure 7). Based on their study, the ammonia capture ratio achieved was 65.2%, which is considered low since the concentration of feed wastewater is low and there are a limited number of stages in HFMC. Their attempt to employ a recirculation mode in HFMC and high ammonia concentration as feed waste has successfully achieved >99% of the ammonia capture ratio, with C_NH_4_^+−^N_ in wastewater decreased less than 10 mg/L. They reported that the energy consumption for the hybrid system is economically reliable since the total processing cost of the hybrid system ($−1.07/ton feed) is lower than that of a single HFMC system ($2.68/ton feed), which was around 111.26 kJ/mol NH_4_^+−^N.

Naidu et al. [46] described the hybrid system of MD with other conventional processes, such as crystallizer, adsorption, forward osmosis, bioreactor and reversed electrodialysis (RED) in resource recovery treatment. This includes nutrient recovery, energy recovery, nitrogen recovery and recover of other valuable organic compounds, including salt. To enhance the MD performance, the physical characteristic of the membrane itself should be improved via modification of membrane fabrication and integration of MD with other processes which can escalate the overall system performance. For example, highly hydrophobic membranes reduce the membrane wetting tendency, whereas thin membranes with opened porous support layers contribute to flux improvement. The geometric structure of the support layer also plays an important role in increasing the flux [47].

Ferrari et al. [48] demonstrated a pilot-scale plant consisting of submerged FO membranes for treating raw municipal wastewater concentration. They studied the membrane performance, fouling behavior and concentration of the wastewater compound with NaCl and MgCl_2_ as draw solutions. When tested with real wastewater, the submerged membrane exhibited excellent resistance to fouling with a draw solution of 11.7g Na/L, water and reverse salt fluxes up to 5.1 ± 1.0 L /m^2^ h and 4.8 ± 2.6 g/m^2^ h were observed. With the achievement of total and soluble chemical oxygen demand concentration factors of 2.47 ± 0.15 and 1.86 ± 0.08, its suitability for application on municipal or other types of complex wastewater is foreseen.

Volpin et al. [45] combined FO and MD systems for the treatment of human urine. When a single MD system was used, the potential of membrane wetting due to the presence of detergent in urine content and permeation of ammonia toward produced water was the main concern [49]. When treated with high urine concentration through the dewatering process, the need of draw solution regeneration would incur additional costs to the system [50]. In this study, the membrane thickness was found to be inversely proportional to the NH_2_ mass transfer and the mass transfer was expected to be low when PTFE membranes were used. It was found that the PTFE membrane plays the main role in reducing nitrogen leakage to the permeate solution during the recovery of the draw solution via MD. Since PTFE possesses higher porosity and thickness over PVDF membranes, PTFE membranes were expected to produce higher water flux and lower NH_3_ flux. It was observed that the nitrogen rejection was high during stored urine filtration in the FO system compared to synthetic fresh urine.

## 4. Challenges and Future Perspective 

Membrane technology is not new, but in the context of wastewater treatment, the technology is still uncommon, as most industries prefer to use conventional technology rather than advanced technology. A few challenges must be considered before pursuing membrane technology, particularly the hollow fiber membrane to be adopted by industries. The limitations come from the membrane availability in the market [51], membrane fouling [52,53,54], technical issues and the cost aspect [51,55].

### 4.1. Membrane Availability

The availability of suitable membranes with the desired performances appropriate for an intended application is the main concern. Although a wide range of membrane products are available in the market, the product is only suited for limited applications as highlighted by Yalcinkaya et al. [56]. This could be the limiting factor in the application of that technology on the go. For example, if the purpose is to use the membrane in the primary treatment process, such as removing total suspended solid (TSS) from the wastewater, the microfiltration (MF) membrane is sufficient for that. In this case, any MF membrane can be purchased online or directly from the supplier. The problem will occur whenever the purpose of the treatment process is to further remove the chemical oxygen demand (COD), biological oxygen demand (BOD) as well as other contaminants present in the wastewater. In this case, cutting-edge membrane technologies, such as nanofiltration (NF), are needed to administer the job. 

At this stage, it is difficult to find a suitable NF membrane for the process on the market. The commercial NF membranes cannot be used for any type of wastewaters as everyone acknowledges that the components and concentrations of pollutants in industrial wastewater are different among industries and hard to define within a specific range. Huang et al. [57] suggested that an ideal membrane should be designed to have high permeability with a steady flow, adequate mechanical strength, durability and excellent chemical stability. In other words, specific or custom-made membranes must be designed based on the given situation. This is aimed to prolong the membrane lifespan and reduce the waste of the membrane.

### 4.2. Membrane Surface Modification

Surface modification is currently at the forefront of membrane research. Various functional inorganic nanomaterials have been applied for the surface modification of polymeric membranes in which the resultant nanocomposite membranes are rendered with synergistic effects to heighten the separation performances. In terms of hollow fiber membrane modification, it is generally observed that the surface modification techniques are more tedious compared to their flat sheet counterparts, mainly due to the setup of modification procedures. While the surfaces of flat sheet membranes can be feasibly subjected to post-fabrication modifications, similar modification may be limited, especially if the modification is intended for the internal surface of the hollow fiber membranes. 

To address this issue, the incorporation of functional nanomaterials during the dope preparation stage becomes more common and practical for the preparation of hollow fiber nanocomposite membranes. Over the last decade, tremendous efforts have been made in the development of ceramic membranes, especially the exploration of green and cheap materials for ceramic membrane development. The applications of ceramic membranes have also expanded with the wider applications of relatively new membrane processes, such as forward osmosis and membrane distillation. The emergence of new contaminants has also prompted the use of more robust ceramic membranes to render more reliable separation performances. The exploration of ceramic membranes for wastewater treatment is expected to grow as an interesting alternative in this field. 

Lately, 3D printing has become a new enabling tool for the development of polymeric membranes in which the technique has been used for the preparation of polymer membrane support and as a technique for interfacial polymerization. With the precise control of fabrication parameters through the 3D printing technique, it is expected that the intrinsic properties of the hollow fiber membrane can be well-tailored. The previously mentioned issue related to membrane modification may also be resolved with the advent of a 3D printing technique in which the surface modifying agent can be precisely introduced on any parts of the hollow fiber membranes. The inception of 3D printing technology in membrane development and processes are expected to reduce both capital and operational cost due to the more energy-efficient design, ease of maintenance and low energy demand during membrane manufacturing. However, it is also worth mentioning that this area of research is still in its infancy stage, with several limitations still hampering the adoption of this technology for large-scale implementation. One significant bottleneck is the economic concern as 3D printing still loses out with regard to the material consumption costs when compared with the materials used in conventional phase inversion and electrospinning techniques. 

Technically, the resolution of the 3D printer is another important consideration. The resolutions required for the membrane fabrication or modification are subject to the intended use of the membranes. In general, the cost of the 3D printer increases with the increasing resolution, which makes this approach still economically unfavorable, especially for nanometer resolutions required for RO application. Nevertheless, it is optimistic that the price of 3D printers will reduce in the coming years with the technological advancements made in this field. 

### 4.3. Technology Aspect and Cost

To be practically used in industrial sectors, it is important to note that technology transfer is necessary during technological changes from the conventional to the advanced treatment method. As pointed out by Li et al. [58], the great challenge in the commercialization aspect is to manage the technological risk after the hollow fiber membrane module is up-scaled following the customer demand that directly affects the local hydrodynamic conditions in the membrane module. They provide an example of how the size of commercial hollow fiber membrane modules has increased by up to 30 cm in diameter and 2.5 m in length just to fulfil the industrial demand. 

In practice, increasing the membrane module will increase the membrane’s tendency to fouling and unstable system operation. The important point that should be taken seriously by the manufacturer or developer of the membrane technology for wastewater treatment is to explain and train the in-house technical persons who take charge of the treatment process to fully understand the membrane treatment process. This not only includes the start/stop process but also how to troubleshoot the process in case of issues during processing. 

Finally, and most importantly, the issue of costs. All industry sectors are aiming for high profit, and the decision to accept membrane technology will be scrutinized thoroughly before finalization. For hollow fiber membrane technology to be accepted by industry executives, the cost of the membrane must be lower than current technology. The energy consumption to operate the membrane process must be lower or within an acceptable range to avoid unnecessary losses. The company may be interested in the performance of the membrane technology, but their main concern is the initial investment cost, maintenance cost and labor and utility costs. As reported by Chia et al. [30], capital costs represent a significant portion (>60%) of the total cost of the PRO, while operational and maintenance costs remain a small part. It is important to note that the capital cost includes not only membrane modules, but also associated pumps, monitoring equipment, fittings and piping [38]. 

## 5. Summary

In a nutshell, membrane technology has been immensely applied and researched in diverse applications, including wastewater treatment and resource recovery. Membrane technology, such as FO, PRO, MD and HFMC have demonstrated huge potential in wastewater treatment and resource recovery since their ability to integrate with other processes exceeded the performance of a single process, thus contributing to lesser capital cost. The modification of the lumen surfaces of hollow fiber membranes via dual-layer membranes has shown potential in treating oily wastewater [59], and by achieving optimal fiber shape that can maximize membrane surface and increase mass transfer, hollow fiber membranes can be utilized as membrane oxygenators in the medical field. Although membrane fouling is the ultimate problem, the approach of combined cleaning method and membrane modification may resolve this problem with an in-depth investigation of real-time fouling behavior and monitoring of fouling mechanism on the membrane surface. More comprehensive studies on a large scale that also consider economic, environmental and technical constraints are needed for this technology to become viable in diverse applications in the future. 

## Figures and Tables

**Table 1:** Applications of hollow fiber membrane in various wastewater treatments.

Applications	Membrane Material	Technology	Feed Solution	Results	Ref.
Municipal domestic wastewater	PP	HFMC	Synthetic human urine	Ammonia recovery efficiency varied from 88.47% to 90.90%	[1]
Domestic wastewaters	PVDF	UF	Domestic wastewaters	60% cake removal	[18]
Synthetic wastewater	PVDF	UF	Synthetic surface wastewater	35–50% reduction in membrane fouling	[19]
Oily wastewater	PVDF	UF	Oily wastewater	70.48 L/m^2^ h permeate flux, 99.7% of oil removal	[20]
Oily wastewater	PVDF	FO-MD	Oily wastewater	>90% feed water recovery	[21]
Gas field wastewater	PES	FO	Mixture of gas field produced water	Reduce the process water volume by 50% at high average water flux of 15.6 L/m^2^ h	[22]
Shale gas wastewater	PVDF, PE, PP	DCMD	Real shale gas wastewater from oil and gas field	13.6% to 27.7% flux reduction, achieve 50% recovery ratio	[23]
Radioactive wastewater	PP	MD	Simulated radioactive wastewater	Removal efficiency >99.67%	[24]
Oilfield wastewater, Seawater	PVDF	MD	Simulated seawater and actual oilfield produced water	Almost 100% of permeate water recovery, 99.9% of salt rejection	[25]
Domestic wastewater	PP	HFMC	High rate activated sludge effluent	The ammonia recovery ratio >98%.	[26]
Seawater, wastewater	PES, Polyamide	FO, PRO	Seawater brine, wastewater brine	Achieve water flux of 40.3 L/m^2^ h (FO), achieve power density as high as 10.6 W/m^2^ (PRO)	[27]
Domestic wastewater	PVDF	HFMC	Synthetic human urine	Production of high-quality fertilizers	[28]

**Table 2:** Recent development in wastewater treatment using hollow fiber membrane for wastewater treatment and resource recovery.

Application	Membrane and Categories	Remarks	Ref.
Recovery of N-P compound fertilizer from human urine	Polypropylene HF (Accurel Q3/2, Membrana Germany) & submerged HFMC	30 hollow fibers, 32 cm length, 0.03 m^2^ effective membrane area ↑ H_3_PO_4_ concentration affects the water vapor transfer and fertilizer components N-content (21.29–55.24 g/L) in the range of the commercial products, P_2_O_5_ content (99.41–281 g/L) slightly higher–can be used in the soils/plants with high demand for phosphorus	[1]
Integration of zeolite adsorption and HFMC	Natural clinoptilolite (Z) zeolite and propylene HFMC Liquid-Cel (Membrane–Charlotte, NC, USA)	NH_4_^+^ adsorbs into zeolites very fast when compared with polymeric materials (zeolite particle diffusion coefficient around 3 × 10^−12^ m^2^/s) The ammonia recovery ratio exceeded 98%	[26]
Ammonia recovery from human urine as liquid fertilizers	PVDF HFM (Econity, Korea) and Direct Contact Membrane Distillation (DCMD)	0.0033 m^2^ effective membrane area, ID/OD = 0.77/1.30 mm	[28]
Removal of ammonia from wastewater	Electrospun PVDF-HFP HFM	KOE: 1.35 × 10^−5^ m/s & S_NH_3_/H_2_O_ = 7.58 (pH = 11) higher than commercial membrane Selectivity improved from 6.91 to 9.74 (↑thickness membrane from 55 ± 5 μm to 115 ± 5 μm)	[37]
Osmotic membrane bioreactor (OMBR)	Membrane substrates: PES HFM, PA selective layer: MPD, TMC	Best performance: air-gap 6 cm, water flux 30.2 L/m^2^ h and SRSF 0.13 g/L with 1 M NaCl and DI water as DS and FS.	[38]
Removal of ammonia from wastewater	CMX, AMX, BP-1E (Astrom Corp Japan), HFMC (Pureseaspring Corp., China)	High concentration of simulated wastewater (NH_4_Cl, NH^4+^ -N, 5000 mg/L), NaCl (2000 mg/L), and Na_2_SO_4_ (2000 mg/L)	[39]
Radioactive wastewater treatment	HTI CTA-ES membrane	FS: 20 mg/L CoCl_2_, 20 mg/L SrCl_2_ and 20 mg/L CsCl in DI water, DS: NaCl, Rejection: >97.7% Co(II), >91.1% Sr(II), >65	[40]
Textile wastewater	FO Aquaporin Inside™ membranes	FS: Real textile wastewater, DS: 1 M NaCl, 1 M MgCl_2_, blue dye mixture and green dye mixture NaCl, Rejection: >94% COD, >99% TDS, TSS, Zn^2+^, and SO_4_^2−^, water recovery: 55%	[41]
Removal of Pb(II) from wastewater	Al_2_O_3_-NaA zeolite composite HFM	Tested synthetic wastewater with removal efficiency 99.9% at 0.1 MPa after 12 h filtration, pore size Al_2_O_3_-NaA zeolite CHFM 0.41 nm diameter	[42]
Removal of palladium catalysts from pharmaceutical industry wastewater	Polypropylene HFSLM system (Liqui-Cel, Membrana/Celgard, Charlotte, NC, USA).	FP: raw pharmaceutical, Extractant: Aliquat 336 Highest percentage of extraction and recovery: 99.95% and 88.12%	[43]
Recovery and purification of potato proteins from potato starch wastewater	Integrated PSF-HF (UF) and PSF-IP HF (NF) membrane	Recovery rates UF and& NF membranes reach 93.5% and 84.7% after physical washing and chemical cleaning UF membrane retained 85.62% potato proteins (high MW) in the potato starch wastewater NF membrane rejected 92.1% potato proteins (low MW)	[44]
Extraction of distilled water from human urine	FO: Flat sheet PA-TFC membrane (Toray Chemical Inc., Tokyo, Japan) MD: PVDF (Durapore^®^-GVHP) and PTFE (Fluoropore^®^-FGLP)	With 2.5 M NaCl (DS), FO membranes achieved water flux of 31.5 (FU)–28.7 (SU) L/m^2^ h, a NH^4+^ flux of 0.1 g /NH^4+^m^2^ h and NH_3_ flux of 1.8 g NH_3_/m^2^ h	[45]

SRSF = specific reverse salt flux, KOE = overall mass transfer coefficient of ammonia, SNH_3_/H_2_O (g) selectivity coefficient of the membrane, MW = molecular weight.

## References

[1] Zhang J., Xie M., Tong X., Yang D., Liu S., Qu D., Feng L., Zhang L. (2021). Ammonia capture from human urine to harvest liquid N-P compound fertilizer by a submerged hollow fiber membrane contactor: Performance and fertilizer analysis. Sci. Total Environ..

[2] Barros Ó., Costa L., Costa F., Lago A., Rocha V., Vipotnik Z., Silva B., Tavares T. (2019). Recovery of Rare Earth Elements from Wastewater towards a Circular Economy. Molecules.

[3] Institute for Rare Earths and Metals AG (2021). Rare Earth Elements—Prices. https://en.institut-seltene-erden.de/our-service-2/.

[4] Ibrahim A.N., Wirzal M.D.H., Nordin N.A.H., Halim N.S.A. (2018). Development of Polyvinylidene fluoride (PVDF)-ZIF-8 Membrane for Wastewater Treatment. IOP Conf. Series Earth Environ. Sci..

[5] Fu F., Wang Q. (2011). Removal of heavy metal ions from wastewaters: A review. J. Environ. Manag..

[6] Kurniawan T.A., Chan G.Y.S., Lo W.-H., Babel S. (2006). Physico–chemical treatment techniques for wastewater laden with heavy metals. Chem. Eng. J..

[7] Ezugbe E.O., Rathilal S. (2020). Membrane Technologies in Wastewater Treatment: A Review. Membranes.

[8] Al-Maamari R.S., Sueyoshi M., Tasaki M., Okamura K., Al-Lawati Y., Nabulsi R.Z., Al-Battashi M. (2014). Flotation, Filtration, and Adsorption: Pilot Trials for Oilfield Produced-Water Treatment. Oil Gas Facil..

[9] Synder Filtration (2021). Model Configuration Process of Hollow Fiber Membrane. https://synderfiltration.com/learning-center/articles/module-configurations-process/hollow-fiber-membranes/.

[10] Azizo A.S., Wirzal M.D.H., Bilad M.R., Yusoff A.R.M. Assessment of nylon 6, 6 nanofibre membrane for microalgae harvesting. Proceedings of the AIP Conference Proceedings.

[11] Hube S., Eskafi M., Hrafnkelsdóttir K.F., Bjarnadóttir B., Bjarnadóttir M. (2020). Ásta; Axelsdóttir, S.; Wu, B. Direct membrane filtration for wastewater treatment and resource recovery: A review. Sci. Total Environ..

[12] Kim J.-O., Jung J.-T., Chung J. (2007). Treatment performance of metal membrane microfiltration and electrodialysis inte-grated system for wastewater reclamation. Desalination.

[13] Meena R.A.A., Kannah R.Y., Sindhu J., Ragavi J., Kumar G., Gunasekaran M., Banu J.R. (2019). Trends and resource recovery in biological wastewater treatment system. Bioresour. Technol. Rep..

[14] Gong H., Jin Z., Wang Q., Zuo J., Wu J., Wang K. (2017). Effects of adsorbent cake layer on membrane fouling during hybrid coagulation/adsorption microfiltration for sewage organic recovery. Chem. Eng. J..

[15] Huang B.C., Guan Y.F., Chen W., Yu H.Q. (2017). Membrane fouling characteristics and mitigation in a coagula-tion-assisted microfiltration process for municipal wastewater pre-treatment. Water Res..

[16] Goh P., Wong K., Ismail A. (2022). Membrane technology: A versatile tool for saline wastewater treatment and resource recovery. Desalination.

[17] Rongwong W., Goh K. (2020). Resource recovery from industrial wastewaters by hydrophobic membrane contactors: A review. J. Environ. Chem. Eng..

[18] Thomas Vroman Mechanisms of Hollow-Fiber Membrane Fouling Removal Used in Water Treatment: Critical Backwash Fluxes and Membrane Deformation for an Enhanced Backwash Efficiency. Polymers. Université Paul Sabatier—Toulouse III, 2020. English. https://tel.archives-ouvertes.fr/tel-02978055/document.

[19] Alresheedi M.T., Basu O.D. (2014). Support media impacts on humic acid, cellulose, and kaolin clay in reducing fouling in a sub-merged hollow fiber membrane system. J. Membr. Sci..

[20] Ong C.S., Lau W.J., Goh P.S., Ng B.C., Ismail A.F. (2015). Preparation and characterization of PVDF–PVP–TiO2 composite hol-low fiber membranes for oily wastewater treatment using submerged membrane system. Desalin. Water Treat..

[21] Zhang S., Wang P., Fu X., Chung T.-S. (2014). Sustainable water recovery from oily wastewater via forward osmosis-membrane distillation (FO-MD). Water Res..

[22] Zhao S., Minier-Matar J., Chou S., Wang R., Fane A.G., Adham S. (2017). Gas field produced/process water treatment using forward osmosis hollow fiber membrane: Membrane fouling and chemical cleaning. Desalination.

[23] Cho H., Choi Y., Lee S. (2018). Effect of pretreatment and operating conditions on the performance of membrane distillation for the treatment of shale gas wastewater. Desalination.

[24] Jia F., Yin Y., Wang J. (2018). Removal of cobalt ions from simulated radioactive wastewater by vacuum membrane distillation. Prog. Nucl. Energy.

[25] Zou L., Zhang X., Gusnawan P., Zhang G., Yu J. (2021). Crosslinked PVDF based hydrophilic-hydrophobic dual-layer hollow fiber membranes for direct contact membrane distillation desalination: From the seawater to oilfield produced water. J. Membr. Sci..

[26] Sancho I., Licon E., Valderrama C., de Arespacochaga N., López-Palau S., Cortina J.L. (2017). Recovery of ammonia from domestic wastewater effluents as liquid fertilizers by integration of natural zeolites and hollow fibre membrane contactors. Sci. Total Environ..

[27] Chou S., Wang R., Shi L., She Q., Tang C., Fane A.G. (2012). Thin-film composite hollow fiber membranes for pressure retarded osmosis (PRO) process with high power density. J. Membr. Sci..

[28] Damtie M.M., Volpin F., Yao M., Tijing L.D., Hailemariam R.H., Bao T., Park K.-D., Shon H.K., Choi J.-S. (2020). Ammonia recovery from human urine as liquid fertilizers in hollow fiber membrane contactor: Effects of permeate chemistry. Environ. Eng. Res..

[29] Mohanty K., Purkait M.K. (2011). Overview of Membrane Science and Technology. Membrane Technologies and Applications.

[30] Chia W.Y., Chia S.R., Khoo K.S., Chew K.W., Show P.L. (2021). Sustainable membrane technology for resource recovery from wastewater: Forward osmosis and pressure retarded osmosis. J. Water Process Eng..

[31] Alkhudhiri A., Darwish N., Hilal N. (2012). Membrane distillation: A comprehensive review. Desalination.

[32] Lu K.J., Zuo J., Chang J., Kuan H.N., Chung T.-S. (2018). Omniphobic Hollow-Fiber Membranes for Vacuum Membrane Distillation. Environ. Sci. Technol..

[33] ScienceDeshmukh A., Boo C., Karanikola V., Lin S., Straub A.P., Tong T., Warsinger D.M., Elimelech M. (2018). Membrane distillation at the water-energy nexus: Limits, opportunities, and challenges. Energy Environ. Sci..

[34] Qi Z., Cussler E. (1985). Microporous hollow fibers for gas absorption: I. Mass transfer in the liquid. J. Membr. Sci..

[35] Darestani M., Haigh V., Couperthwaite S., Millar G., Nghiem L. (2017). Hollow fibre membrane contactors for ammonia recovery: Current status and future developments. J. Environ. Chem. Eng..

[36] Rangwala H.A. (1996). Absorption of carbon dioxide into aqueous solutions using hollow fiber membrane contactors. J. Membr. Sci..

[37] Ma X., Li Y., Cao H., Duan F., Su C., Lu C., Chang J., Ding H. (2019). High-selectivity membrane absorption process for recovery of ammonia with electrospun hollow fiber membrane. Sep. Purif. Technol..

[38] Lim S., Tran V.H., Akther N., Phuntsho S., Shon H.K. (2019). Defect-free outer-selective hollow fiber thin-film composite membranes for forward osmosis applications. J. Membr. Sci..

[39] Yan H., Wu L., Wang Y., Irfan M., Jiang C., Xu T. (2020). Ammonia capture from wastewater with a high ammonia nitrogen concentration by water splitting and hollow fiber extraction. Chem. Eng. Sci..

[40] Liu X., Wu J., Hou L., Wang J. (2020). Fouling and cleaning protocols for forward osmosis membrane used for radioactive wastewater treatment. Nucl. Eng. Technol..

[41] Korenak J., Hélix-Nielsen C., Bukšek H., Petrinić I. (2019). Efficiency and economic feasibility of forward osmosis in textile wastewater treatment. J. Clean. Prod..

[42] Zhu L., Ji J., Wang S., Xu C., Yang K., Xu M. (2018). Removal of Pb(II) from wastewater using Al2O3-NaA zeolite composite hollow fiber membranes synthesized from solid waste coal fly ash. Chemosphere.

[43] Sunsandee N., Phatanasri S., Pancharoen U. (2021). Separation of homogeneous palladium catalysts from pharmaceutical industry wastewater by using synergistic recovery phase via HFSLM system. Arab. J.Chem..

[44] Li H., Zeng X., Shi W., Zhang H., Huang S., Zhou R., Qin X. (2020). Recovery and purification of potato proteins from potato starch wastewater by hollow fiber separation membrane integrated process. Innov. Food Sci. Emerg. Technol..

[45] Volpin F., Chekli L., Phuntsho S., Ghaffour N., Vrouwenvelder H., Shon H.K. (2019). Optimisation of a forward osmosis and membrane distillation hybrid system for the treatment of source-separated urine. Sep. Purif. Technol..

[46] Naidu G., Tijing L., Johir M.A.H., Shon H., Vigneswaran S. (2020). Hybrid membrane distillation: Resource, nutrient and energy recovery. J. Membr. Sci..

[47] Zhang J., Dow N., Duke M., Ostarcevic E., Li J.-D., Gray S. (2010). Identification of material and physical features of membrane distillation membranes for high performance desalination. J. Membr. Sci..

[48] Ferrari F., Pijuan M., Rodriguez-Roda I., Blandin G. (2019). Exploring Submerged Forward Osmosis for Water Recovery and Pre-Concentration of Wastewater before Anaerobic Digestion: A Pilot Scale Study. Membranes.

[49] Tun L.L., Jeong D., Jeong S., Cho K., Lee S., Bae H. (2016). Dewatering of source-separated human urine for nitrogen recovery by membrane distillation. J. Membr. Sci..

[50] Volpin F., Chekli L., Phuntsho S., Cho J., Ghaffour N., Vrouwenvelder J.S., Shon H.K. (2018). Simultaneous phosphorous and nitrogen recovery from source-separated urine: A novel application for fertiliser drawn forward osmosis. Chemosphere.

[51] Alsebaeai M.K., Ahmad A.L. (2020). Membrane distillation: Progress in the improvement of dedicated membranes for enhanced hydrophobicity and desalination performance. J. Ind. Eng. Chem..

[52] Choudhury M.R., Anwar N., Jassby D., Rahaman M.S. (2019). Fouling and wetting in the membrane distillation driven wastewater reclamation process—A review. Adv. Colloid Interface Sci..

[53] Fortunato L., Jang Y., Lee J.-G., Jeong S., Lee S., Leiknes T., Ghaffour N. (2018). Fouling development in direct contact membrane distillation: Non-invasive monitoring and destructive analysis. Water Res..

[54] Muhamad N., Mokhtar N.M., Naim R., Lau W., Ismail A. (2019). A Review of Membrane Distillation Process: Before, During and After Testing. Int. J. Eng. Technol. Sci..

[55] Kamali M., Suhas D., Costa M.E., Capela I., Aminabhavi T.M. (2019). Sustainability considerations in membrane-based technologies for industrial effluents treatment. Chem. Eng. J..

[56] Yalcinkaya F., Boyraz E., Maryska J., Kucerova K. (2020). A Review on Membrane Technology and Chemical Surface Modification for the Oily Wastewater Treatment. Materials.

[57] Huang Y., Xiao C., Huang Q., Liu H., Zhao J. (2021). Progress on polymeric hollow fiber membrane preparation technique from the perspective of green and sustainable development. Chem. Eng. J..

[58] Li X., Mo Y., Li J., Guo W., Ngo H.H. (2017). In-situ monitoring techniques for membrane fouling and local filtration characteristics in hollow fiber membrane processes: A critical review. J. Membr. Sci..

[59] Yaacob N., Goh P.S., Ismail A.F., Nazri N.A.M., Ng B.C., Abidin M.N.Z., Yogarathinam L.T. (2020). ZrO_2_-TiO_2_ Incorporated PVDF Dual-Layer Hollow Fiber Membrane for Oily Wastewater Treatment: Effect of Air Gap. Membranes.

